# Chemical Synthesis and Structure–Activity Relationship Studies of the Coagulation Factor Xa Inhibitor Tick Anticoagulant Peptide from the Hematophagous Parasite *Ornithodoros moubata*

**DOI:** 10.3390/biomimetics9080485

**Published:** 2024-08-12

**Authors:** Vincenzo De Filippis, Laura Acquasaliente, Andrea Pierangelini, Oriano Marin

**Affiliations:** 1 Laboratory of Protein Chemistry & Molecular Haematology, Department of Pharmaceutical and Pharmacological Sciences, School of Medicine, University of Padova, Via F. Marzolo 5, 35131 Padua, Italy; laura.acquasaliente@unipd.it (L.A.); andrea.pierangelini@unipd.it (A.P.); 2 Department of Biomedical Sciences, School of Medicine, University of Padova, Via Trieste 75, 35121 Padua, Italy; oriano.marin@unipd.it

**Keywords:** natural anticoagulants, tick anticoagulant peptide, non-coded amino acids, protease inhibitors, HDX-MS, peptide synthesis, molecular recognition, coagulation

## Abstract

Tick Anticoagulant Peptide (TAP), a 60-amino acid protein from the soft tick *Ornithodoros moubata*, inhibits activated coagulation factor X (fXa) with almost absolute specificity. Despite TAP and Bovine Pancreatic Trypsin Inhibitor (BPTI) (i.e., the prototype of the Kunitz-type protease inhibitors) sharing a similar 3D fold and disulphide bond topology, they have remarkably different amino acid sequence (only ~24% sequence identity), thermal stability, folding pathways, protease specificity, and even mechanism of protease inhibition. Here, fully active and correctly folded TAP was produced in reasonably high yields (~20%) by solid-phase peptide chemical synthesis and thoroughly characterised with respect to its chemical identity, disulphide pairing, folding kinetics, conformational dynamics, and fXa inhibition. The versatility of the chemical synthesis was exploited to perform structure–activity relationship studies on TAP by incorporating non-coded amino acids at positions 1 and 3 of the inhibitor. Using Hydrogen–Deuterium Exchange Mass Spectrometry, we found that TAP has a remarkably higher conformational flexibility compared to BPTI, and propose that these different dynamics could impact the different folding pathway and inhibition mechanisms of TAP and BPTI. Hence, the TAP/BPTI pair represents a nice example of divergent evolution, while the relative facility of TAP synthesis could represent a good starting point to design novel synthetic analogues with improved pharmacological profiles.

## 1. Introduction

The main source of natural anticoagulants is represented by blood-feeding parasites (e.g., leeches, ticks, mosquitos, and nematodes) that, to overcome host response systems (i.e., activation of blood coagulation in response to vascular damage), produce highly potent and selective small protein inhibitors of the coagulation proteases, especially thrombin and activated coagulation factor X (fXa) [1,2,3,4,5]. Both thrombin and fXa are chymotrypsin-like serine proteases playing a pivotal role in haemostasis. Thrombin is the final effector protease of the coagulation cascade, as it proteolytically converts soluble fibrinogen into insoluble fibrin aggregates and activates by proteolysis type-1 protease-activated receptor on the surface of platelets, which become bio-adhesive and agglutinate to form, together with fibrin, the final thrombus structure [6]. Interestingly, thrombin is generated after proteolysis of prothrombin zymogen by fXa in the prothrombinase complex on the surface of activated platelets [7]. Given the unique position of fX in the coagulation pathway, this protease has emerged as an attractive target for the development of new anticoagulants of both synthetic [8] and natural origin [5].

Among novel anticoagulants of natural origin, Tick Anticoagulant Peptide (TAP), a 60-amino acid small protein extracted from the soft tick *Ornithodoros moubata* [9], was the first direct inhibitor of fXa to be investigated. Noteworthy, TAP inhibits in a highly specific manner free fXa (K_i_ = 180 pM) [10] and its potency is even increased by >30-fold toward factor Xa assembled within prothrombinase complex (K_i_ = 5.3 pM) [11]. The inhibition of fXa is considered safer than the blockage of thrombin, as the former does not preclude the generation of sufficient amounts of thrombin to effect haemostasis [12]. The antithrombotic efficacy of TAP has been assessed in a number of in vivo animal models and recombinant TAP was indeed found to be more effective than heparin (i.e., a sulphurylated natural glycosaminoglycan amplifying the blockage of thrombin and fXa by physiological inhibitors) and was at least as effective as hirudin (i.e., a small protein secreted by the salivary glands of the leech *Hirudo medicinalis*) but with reduced bleeding [13,14,15,16,17,18,19]. More recently, fused protein constructs containing both TAP and single-chain antibodies, selectively targeting platelet-activated α_IIb_β_3_ integrin, allowed the inhibition of excessive fX activation at sites of thrombus formation via the localization of the antithrombotic activity of TAP without compromising haemostasis [19,20,21,22]. This dual-pathway inhibition strategy allowed for the uncoupling of therapeutic efficacy from the deleterious bleeding effect and was proven to achieve equivalent in vivo efficacy to currently available antiplatelet agents or Direct Oral Anticoagulants (DOACs) (i.e., small-molecule inhibitors of thrombin or fXa), either alone or in combination, at much lower doses, therefore dramatically reducing the overall bleeding risk [23].

**Figure 1:**
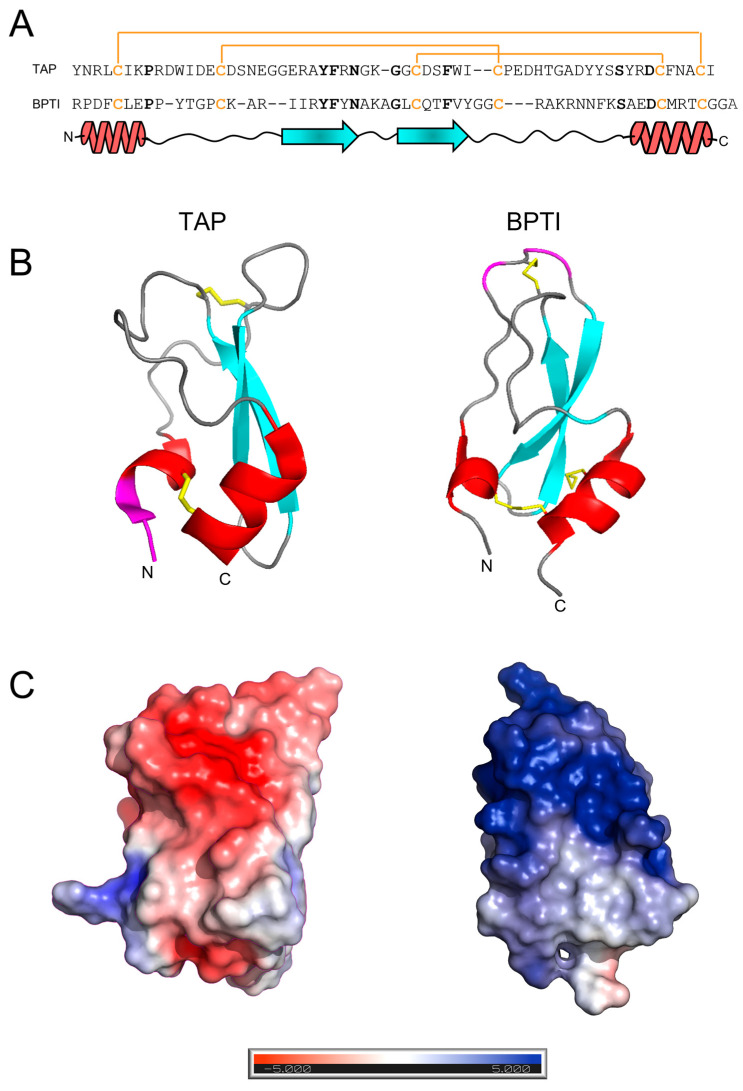
The amino acid sequence and structural similarity of TAP and BPTI. (**A**) The amino acid sequence alignment of TAP and BPTI: Conserved residues are indicated in bold. The disulphide bond topology is conserved in the two inhibitors and indicated by plain lines (orange). The secondary structure alignment of TAP and BPTI is also reported. (**B**) The three-dimensional structure of TAP and BPTI: Ribbon drawing representations are based on the best representative NMR conformers of TAP (1tcp.pdb) and BPTI (1pit.pdb). Helical regions are coloured in red, β-strands are in cyan, while segments of irregular structure are in light grey. The regions involved in trypsin or fXa binding are shown in magenta. N- and C-termini are also indicated. (**C**) The surface electrostatic potentials of TAP and BPTI: The orientation of the two inhibitors are as in panel B. The surface is coloured according to the electrostatic potential (blue, positive; red, negative) and expressed as kJ/(mol·q), as indicated. Calculations were performed using the APBS programme, run on the coordinates of the best representative conformers in the NMR structure of TAP and BPTI. Protein structure images were generated using the PyMOL ver. 1.3 Molecular Graphics System.

Beyond its biological function and pharmacological potential, the three-dimensional structure of TAP closely resembles that of Bovine Pancreatic Trypsin Inhibitor (BPTI), the prototype of the Kunitz family of protein protease inhibitors (Figure 1B), with which TAP shares a characteristic secondary structure content (i.e., a β-hairpin followed by a C-terminal α-helix) and disulphide bond topology [2]. Despite the structural similarity existing between TAP and BPTI, the two inhibitors display only weak sequence identity (~24%) that becomes negligible (~14%) if the conservation of disulphide bonds is not considered (Figure 1C). Furthermore, TAP and BPTI follow different pathways in oxidative disulphide folding reaction to achieve the final native structure [24] and have remarkably different thermal stabilities [25,26]. More importantly, the two inhibitors have very different selectivity toward serine proteases [27] and exploit totally different mechanisms of action for inhibiting their corresponding target proteases [9,10].

With this in mind, here we chemically synthesised by stepwise solid-phase peptide synthesis disulphide-folded fully active wild-type TAP 1–60. The conformational and dynamic properties of TAP were investigated by optical spectroscopic methods (i.e., circular dichroism and fluorescence) and Hydrogen–Deuterium Exchange Mass Spectrometry (HDX-MS), in comparison with BPTI. Taking advantage of the versatility of chemical synthesis, structure–activity relationship (SAR) studies were conducted by inserting non-coded amino acids (i.e., *p*-amino-Phe, pyridyl-Ala, *p*-aminomethyl-Phe, *p*-guanido-Phe, and β-naphthyl-Ala) in the TAP structure at positions 1 and 3.

## 2. Materials and Methods

### 2.1. Reagents

N^α^-Fmoc-protected amino acids, solvents, and reagents for peptide synthesis were purchased from Applied Biosystems (Foster City, CA, USA) or from Advanced Chemtech (Louisville, KY, USA). TFA, β-mercaptoethanol, polyethylene glycol (PEG) 8000, and deuterated water (isotopic purity >99.9% in D_2_O) were acquired from Fluka (Basel, Switzerland). Factor Xa from human plasma, bovine pancreas trypsin and chymotrypsin, and BPTI were purchased from Calbiochem (La Jolla, CA, USA). The Arg-Gly-Arg-*p*-nitroanilide peptide substrate was obtained from Bachem AG (Bubendorf, Switzerland). All other reagents, buffers, and organic solvents were of analytical grade and were from Merck (Darmstadt, Germany).

### 2.2. Peptide Synthesis

Wild-type TAP and mutated analogues were synthesised by the solid-phase method using the 9-fluorenylmethyloxycarbonyl (Fmoc)/*t*-butyl (*t*Bu) strategy previously detailed [28]. Synthesis was carried out in two sequential steps on an Ile-derivatized (0.92 mmol/g of resin; 272 mg) p-alkyl benzyl ester polystyrene resin (1% divinylbenzene cross-linked). In the first step, the peptide chain corresponding to sequence 5–60 was assembled stepwise using an automated PS3 model synthesiser from Gyros Protein Technologies International (Tucson, AZ, USA) or with a model 431 synthesiser from Applied Biosystems (Foster City, CA, USA). Standard coupling reactions and the protection of side-chain reactivity were performed. The removal of N^α^-Fmoc protecting groups was achieved by 15 min treatment with 20% piperidine in N-methylpyrrolidone. After the assembly of the peptide chain 5–60 was completed, 2.636 g of protected peptidyl resin was obtained.

For each analogue, coupling of the remaining four amino acids was performed by manual peptide synthesis with 20 µmoles of protected 5–60 peptidyl resin. Cleavage of the peptide from the resin and side-chain deprotection was simultaneously achieved by 90 min treatment with the following mixture: TFA (80%), crystalline phenol (3.5% *w*/*v*), thioanysole (6%), ethandithiol (6%), triisopropylsilane (1%), and deionized water (3.5%). After filtration of the resin and precipitation with ice-cold diethyl ether, the crude peptide was dissolved in 0.1% aqueous TFA and fractionated by RP-HPLC on a Vydac (Grace-Vydac, Hesperia, CA, USA) C18 column (4.6 × 250 mm, 5 µm particle size), eluted with a linear acetonitrile-0.1% TFA from 29 to 33% for 30 min at a flow rate of 0.8 mL/min. The material eluted in correspondence with the major chromatographic peak was collected and its chemical identity established by high resolution mass spectrometry (HR-MS) using a model Mariner Esi-TOF (PerSeptive BioSystems, Framingham, MA, USA) or a Waters (Milford, MO, USA) Xevo G2-S Q-TOF mass spectrometer. For preparative purposes, the crude peptide material was purified by semi-preparative RP-HPLC. The purified peptide with the six Cys residues in the reduced state was dissolved in 0.05% aqueous TFA, containing arginine hydrochloride, and added dropwise to the refolding buffer, constituted by 0.1 M Tris-HCl, pH 8.4, to a final protein concentration of 0.5 mg/mL and 0.5 M arginine. TAP was allowed to fold for 24 h under air oxidation conditions in the presence of 250 µM β-mercaptoethanol [29]. The refolded disulphide-oxidised TAP analogues were purified by semi-preparative RP-HPLC and analysed by HR-MS (see Supplementary Table S1).

### 2.3. Assignment of Disulphide Pairings

The correctness of the disulphide topology in refolded synthetic TAP was established by combining enzymatic fingerprint analysis and HR-MS. Purified, refolded TAP 1–60 (0.8 mg/mL) in 0.1 M NaHCO_3_, pH 7.8, was added to immobilised trypsin and α-chymotrypsin Sepharose-4B gel (Merck) in an Ultrafree^TM^ polypropylene tube (Millipore, Bedford, MA, USA). The proteolysis reaction was carried out at 40 °C for 4 h. After centrifugation, the supernatant was acidified and fractionated by analytical RP-HPLC. The chemical identity of the peptides eluted from the column was established by HR-MS (Supplementary Table S2).

### 2.4. Disulphide Folding Kinetics

Disulphide-oxidised TAP (0.5 mM) was incubated for 2 h in the dark at 37 °C in 0.5 M Tris-HCl buffer, pH 8.4, containing 0.15 M DTT, 1 mM ethylenediaminetetraacetate disodium salt and 5 M guanidinium hydrochloride (Gdn-HCl). The reduction mixture was desalted on a HiTrap fast-flow desalting column (Cytiva, Marlborough, MA, USA), eluted with the refolding buffer (0.1 M Tris-HCl buffer, pH 8.4), at a flow rate of 2 mL/min, and recorded the absorbance of the effluent at 280 nm. The reduced TAP species was eluted in correspondence with the void volume and its concentration determined by measuring the UV absorbance of the solution at 280 nm (ε_280nm_ = 17,780 M^−1^·cm^−1^). TAP folding (140 µM) was allowed to proceed for 24 h at 25 °C in the presence of 250 µM β-mercaptoethanol. At time intervals, aliquots (20 µg) were taken, quenched with 4% aqueous TFA (40 µL), and fractionated with a (4.6 × 250 mm, 5 µm particle size) Vydac (Grace-Vydac, Hesperia, CA, USA) Cl8 analytical column, eluted with a linear acetonitrile-0.1% TFA gradient from 20 to 40% for 30 min at a flow rate of 0.8 mL/min and connected to a model 1500 Jasco (Tokyo, Japan) HPLC system. The absorbance of the effluent was monitored at 226 nm.

### 2.5. Spectroscopic Measurements

Protein concentration was determined spectrophotometrically on a Jasco (Tokyo, Japan) double beam V-630 UV/Vis spectrophotometer using the molar absorptivity values reported in Supplementary Table S1.

Circular dichroism (CD) spectra were recorded on a Jasco (Tokyo, Japan) J-810 spectropolarimeter equipped with a Jasco Peltier model ETC-273T temperature control system [30,31]. All measurements were performed in phosphate-buffered saline (PBS), pH 7.8, at 25 ± 0.1 °C. The final spectra resulted from the average of four accumulations, after baseline subtraction.

Fluorescence measurements were performed at 25 ± 0.1 °C in PBS using a Jasco (Tokyo, Japan) FP-6500 spectrofluorometer, equipped with a Jasco Peltier model ETC-273T temperature control system [30,31]. Spectra were subtracted for the corresponding baselines.

### 2.6. Hydrogen–Deuterium Exchange Mass Spectrometry (HDX-MS)

HDX-MS measurements were performed using a Xevo G2S Q-TOF (Waters, Milford, MO, USA) mass spectrometer, equipped with (i) a standard electrospray ionisation source, (ii) an Acquity M-class UPLC (Waters, Milford, MO, USA), (iii) an Automation 2.0 sample workstation (Waters, Milford, MO, USA), and (iv) an HDX PAL autosampler (Leap Technologies, Carrboro, NC, USA), as previously reported [32,33]. Purified TAP (25 μM) was incubated at increasing time points (10 s–2 h) in deuterated buffer (i.e., 20 mM sodium phosphate in 95:5 D_2_O:H_2_O solution, pD 7.4, 150 mM NaCl). For each sample, H/D exchange was quenched at 0 °C by addition of an equal volume of quenching buffer, i.e., 20 mM sodium phosphate in H_2_O, containing 0.5 M Tris(2-carboxyethyl)phosphine hydrochloride and 3M Gdn-HCl, adjusted to pH 2.35. The pH and pD values were measured at 25 °C, using a Mettler-Toledo (Columbus, OH, USA) mod. FiveEasy Plus pH meter.

Global HDX analyses were performed as three technical replicates. For the H/D exchange reaction, an aliquot (2 μL) of each sample was diluted 20-fold in deuterated buffer (20 mM sodium phosphate, 150 mM NaCl, pD 7.43, in 99.9% D_2_O) and was then allowed to exchange from 10 s to 1 h. At each time point, the H/D exchange reaction was quenched at 0 °C by adding an equal volume of quenching buffer, i.e., 0.8% formic acid, to give a final pH of 2.52. The quenched samples were immediately injected onto a MassPrep Micro Desalting column (Waters, Milford, MO, USA) thermostated at 0 °C, connected to a Waters (Milford, MO, USA) M-class UPLC system and to a Xevo G2-S Q-TOF mass spectrometer (Waters, Milford, MO, USA. The column was equilibrated for 3.5 min at a flow rate of 100 μL/min in 95% eluent A (0.23% formic acid in water) and 5% eluent B (0.23% formic acid in acetonitrile), and eluted with a gradient of 5% to 50% of eluent B for 1 min and of 50% to 70% of eluent B for 2 min, at a flow rate of 75 μL/min. MS spectra of the effluent from the column were acquired over the range of 50 to 2000 *m*/*z*. The instrument configuration settings were as follows: capillary voltage 1.5 kV, sampling cone 40 V, source temperature 120 °C, and desolvation temperature 280 °C. Leu-enkephalin was continuously infused as the reference lock mass. To minimise carry-over effects, a blank was performed after each analysis. The incorporation of deuterium into intact proteins was determined by measuring the relative deuterium uptake (%D) at increasing incubation times according to the expression [34] %D = (m_t_ − m_0_)/(m_100_ − m_0_), where m_t_ is the mass of the protein after the labelling time t, and m_0_ and m_100_ are the masses of the non-deuterated and maximally deuterated protein, respectively. Data are reported as the mean of the three analyses with the error as the standard deviation (±S.D.). Samples for the maximally deuterated controls, used for back-exchange corrections, were prepared as described [35]. Briefly, proteins were lyophilized, resuspended in 7 M Gdn-HCl, and heated at 90 °C for 5 min. After cooling to room temperature, the labelling buffer was added to the denatured protein solution, obtaining a final 95% D_2_O concentration, while the exchange reaction proceeded at 40 °C for 10 min. The samples were then cooled to room temperature and quenched as above. Data were analysed with DynamX ver. 3.0 software (Waters, Milford, MO, USA).

### 2.7. Factor Xa Inhibition Assays

The enzyme inhibition assays were all performed at 25 ± 0.1 °C in TBS (5 mM Tris–HCl, pH 7.4, 0.1% PEG-8000 *w*/*v*) in the presence of 0.2 M NaCl, using a Jasco (Tokyo, Japan) double-beam V-630 spectrophotometer equipped with a Jasco Peltier model PAC-740 temperature control system. Tight-binding inhibition assays [32] were performed by pre-incubating fXa (100 pM) for 40 min with increasing [I] inhibitor concentrations (0–2 nM). The reaction was started by adding the chromogenic substrate RGR-pNA (300 μM) and the release of p-nitroaniline (pNA) was continuously monitored at 405 nm, using a 9920 M^−1^·cm^−1^ molar absorptivity. The relative rate of substrate hydrolysis (v_i_/v_0_) was a function of [I]. The apparent inhibition constant, K_I_^app^, was determined as a fitting parameter by interpolating the data points to the equation describing the tight-binding inhibition model, as detailed elsewhere [34]. The K_I_^app^ value was then converted into the real inhibition constant, K_I_, under the assumption of competitive inhibition and using a K_m_ value of substrate hydrolysis by fXa of 60 ± 1 μM at 25 °C [36].

### 2.8. Computational Methods

Accessible surface area (ASA) calculations were performed using the WHAT IF software [37], available online at the webpage https://swift.cmbi.umcn.nl/servers/html/index.html (accessed on 29 July 2024) and run on the first conformer in the nuclear magnetic resonance (NMR) ensembles of TAP reported in the Protein Data Bank, i.e., 1tap.pdb [25] or 1tcp.pdb [38]. Electrostatic potential calculations were carried out using APBS-PDB2PQR ver. 3.6.1 [39] and BLUUES [40] software available online at the webpage http://protein.bio.unipd.it/bluues/ (accessed on 29 July 2024), using standard parameters. The structure of the synthetic TAP analogues in the fXa-bound state was modelled using the Molecular Operating Environment (MOE) ver. 15 software and the OPLS-AA force field (Chemical Computing Group, Montreal, QC, Canada), run on the crystallographic structure of the TAP-fXa complex (lkig.pdb) [10]. The physicochemical properties of the substituting amino acid side chains were taken from experimentally determined data sets [41,42]. Images were generated with PyMOL ver. 1.3 (DeLano Scientific, San Diego, CA, USA).

## 3. Results

### 3.1. Chemical Synthesis and Disulphide Oxidative Renaturation of Wild-Type TAP

**Figure 2:**
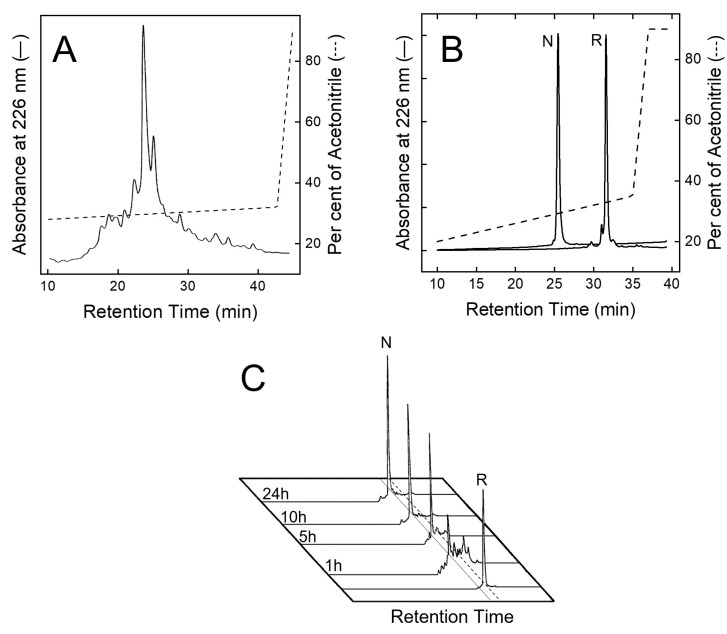
Purification and disulphide oxidative renaturation of TAP. (**A**) RP-HPLC analysis of crude, synthetic TAP after resin cleavage and side chain protecting group removal. (**B**) RP-HPLC analysis of reduced (R) and oxidised (N) TAP after purification by semi-preparative RP-HPLC. (**C**) Kinetics of disulphide oxidative renaturation of TAP. Purified TAP (1 mg/mL) with Cys residues in reduced state (R) was allowed to fold under air oxidation conditions, at pH 8.3 in the presence of β-mercaptoethanol (250 μM) (see text). At fixed time points, aliquots (20 μL) were taken, acid quenched, and fractionated by analytical RP-HPLC to estimate folding yields of native TAP (N).

After peptide chain assembly, the removal of side chain protecting groups, and final release from the solid support, the crude peptide with Cys residues in the reduced form (R-TAP) was fractionated by analytical RP-HPLC (Figure 2A). The peptide material eluted in correspondence with the major chromatographic peak was collected and analysed by HR-MS, yielding a mass value (6984.3 ± 0.2 a.m.u.) in agreement with the theoretical average mass of R-TAP (6984.6 a.m.u.). From Figure 2A, a chromatographic yield of ~28% could be estimated. The reduced species was purified in sufficient amounts by semi-preparative RP-HPLC, lyophilized and subjected to an oxidative disulphide folding reaction. Disulphide bond renaturation was carried out by incubating R-TAP in the refolding buffer, containing 250 μM β-mercaptoethanol and 0.5 M arginine. After a 24 h reaction, a predominant species, hereafter denoted as native TAP (N-TAP), was obtained. The molecular mass of N-TAP (6978.5 ± 0.3 a.m.u.) was six units lower than that of R-TAP, consistent with the formation of the three disulphide bonds present in the native protein. N-TAP was purified by semi-preparative RP-HPLC (Figure 2B), while the correctness of S-S bond topology (see Figure 1A) was established by peptide mass fingerprint analysis with trypsin and chymotrypsin (Supplementary Figure S1 and Table S2).

The kinetics of disulphide bond formation were monitored by RP-HPLC (Figure 2C), showing the progressive accumulation in the first five hours of the faster-eluting N-TAP species, reaching a plateau after 24 h reaction, and with a final recovery >85%. In conclusion, the overall final yield of pure N-TAP was found to be ~20%.

### 3.2. Spectroscopic Characterization of Synthetic Wild-Type TAP

**Figure 3:**
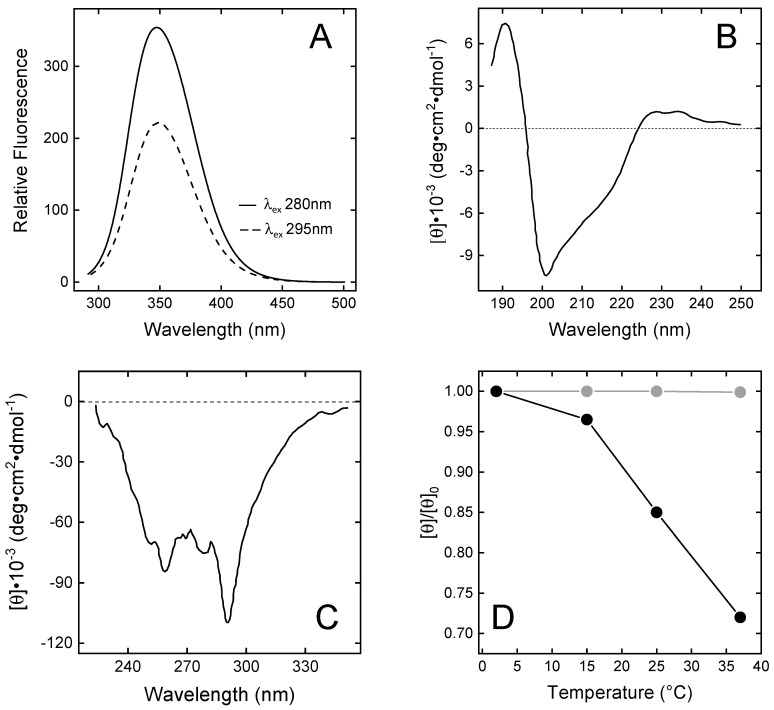
Spectroscopic characterisation of N-TAP. (**A**) Fluorescence spectra of N-TAP. Protein samples (50 µg/mL) were excited at 280 and 295 nm. (**B**,**C**) Far-UV (**B**) and near-UV (**C**) circular dichroism spectra of N-TAP. Spectra were recorded at protein concentration of 0.1 mg/mL and 1 mg/mL in far- and near-UV region, respectively. All measurements were carried out at 25 ± 0.1 °C in PBS, pH 7.4, and resulting spectra were subtracted for corresponding baselines. (**D**) Temperature dependence of the relative ellipticity (θ/θ_0_) of TAP (●) and BPTI (●). Ellipticity values (θ) of TAP and BPTI (1 mg/mL) were recorded at 289 nm as a function of temperature and normalised by the initial value (θ_0_) measured at 2 °C.

The fluorescence spectrum of wild-type N-TAP (Figure 3A) displays a λ_max_ value at 348 nm, after excitation at 280 nm, indicating that the two Trp residues (i.e., Trp11 and Trp37) are both located in a highly polar environment [43]. Accessible surface area (ASA) calculations, run on the NMR solution structure of TAP [25,38], indicate that, whereas Trp37 is indeed highly exposed (>75%) to the polar water solvent, Trp11 is buried in the protein core, in van der Waals contact with the apolar side chains of Phe36, Ile38, and Tyr48. Therefore, Trp11 is expected to emit at much shorter λ_max_ values. Careful inspection of TAP structure, however, confirms that Trp11 is shielded from the solvent (%ASA = 12%) but reveals that the N-H indolyl group is hydrogen-bonded to the carboxylate group of Asp13 nearby, thus explaining the red-shifted emission of TAP (Figure 3A).

The far-UV CD spectrum of N-TAP (Figure 3B) is similar to that previously reported for the recombinant TAP expressed in *E. coli* [25] or *Pichia pastoris* [38], and is typical of a protein containing both mixed α/β secondary structure and a significant amount of irregular structure, consistent with the NMR solution structure of N-TAP [25,38], showing the presence of a twisted two-stranded antiparallel sheet and a C-terminal α-helix. The N-terminal region (residues 2–7) assumes a 3_10_-helical conformation and the remaining regions display a non-periodic/irregular structure.

The near-UV CD spectrum of N-TAP (Figure 3C) displays extensive fine structure, indicating that aromatic amino acids (5Tyr, 3Phe, and 2Trp) are embedded on average into rigid and asymmetric environments in the TAP structure, with a prominent negative band at 289 nm (assigned to the contribution of Trp residues) and the 6 nm spaced bands in the 250–270 nm range (assigned to the contribution of Phe residues) [44]. Next, we comparatively monitored the temperature dependence of the CD signal at the λ_max_ for TAP and BPTI in the temperature range 2–37 °C. The data in Figure 3D clearly indicate that the temperature dependence of the relative ellipticity (θ/θ_0_) for TAP is remarkably higher than for BPTI. At 37 °C, a 30% reduction in θ/θ_0_ for TAP is measured, whereas the same signal remains unchanged with BPTI. These observations suggest that, despite sharing a similar three-dimensional fold, the TAP structure is looser and less stable than that of BPTI.

### 3.3. Probing the Structure and Dynamics of Synthetic TAP by HDX-MS

HDX-MS is an emerging powerful technique in structural biology, useful for investigating protein dynamics and molecular recognition [45,46,47]. HDX-MS basically exploits the intrinsic propensity of backbone amide hydrogens at exposed/flexible sites to exchange more rapidly with deuterium than those hydrogens that are buried in the protein interior or at the ligand–protein interface and therefore will exchange much more slowly [45,46]. HDX can be monitored by recording the time-dependent mass increase of the whole/intact protein (i.e., global exchange analysis) or of short fragments, generated after proteolysis with pepsin (i.e., local exchange analysis).

**Figure 4:**
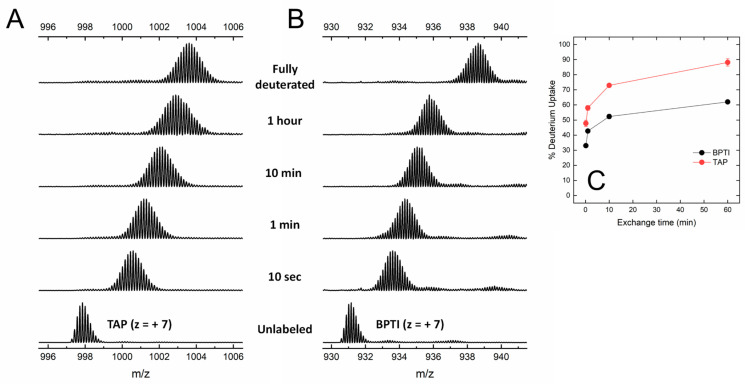
Global HDX-MS analysis of N-TAP and BPTI. (**A**,**B**) Representative traces of global deuterium uptake of N-TAP (**A**) and BPTI (**B**) at increasing H/D exchange times. Proteins (25 μg/mL) were incubated at 25 °C with 95% D_2_O in PBS buffer, pD 7.43, and *m*/*z* spectra were taken at increasing labelling times, as indicated. For both N-TAP (*m*/*z* = 997.875, average value) and BPTI (*m*/*z* = 931.166, average value), multiple charged species at z = +7 were selected for monitoring deuterium uptake. (**C**) Time-course analysis of %D increase in TAP and BPTI, as indicated. Experimental conditions are those reported in panels (**A**,**B**). Data points are average of three different experiments, with error bars as standard deviations (see Methods).

Under the conditions tested, both BPTI and TAP were largely resistant to pepsin hydrolysis, likely because the three S-S bonds present in the structure (Figure 1) impose stereochemical constrains that impaired the polypeptide chain to adapt to and be cleaved by the protease active site. The practical impossibility to run local exchange analysis prompted us to perform HDX-MS measurements in the global exchange mode, to obtain more (albeit cumulative) insights into the main-chain flexibility of TAP and BPTI. The data in Figure 4A,B show that, for both intact TAP and BPTI, the *m*/*z* centroid of the selected multiple-charged species rapidly increases with a burst-phase of %D and then gradually shifts to higher masses. This trend is indicative of an EX2 exchange mechanism, which is typical of natively folded proteins undergoing fast local fluctuations at multiple sites [33,45]. Quantitative data analysis (Figure 4C) indicates that the fraction of hydrogen ions that undergo burst phase exchange with deuterium ions in the dead time of the experiment is remarkably higher for TAP (48%) compared to BPTI (32%), indicating that TAP has a looser/more flexible structure than that of BPTI. Interestingly, the time-dependent increase in %D measured at longer incubation times follows a very similar trend with both TAP and BPTI, consistent with the overall fold similarity of the two proteins (Figure 1).

### 3.4. Inhibition of fXa Amidolytic Activity by Wild-Type Synthetic TAP

**Figure 5:**
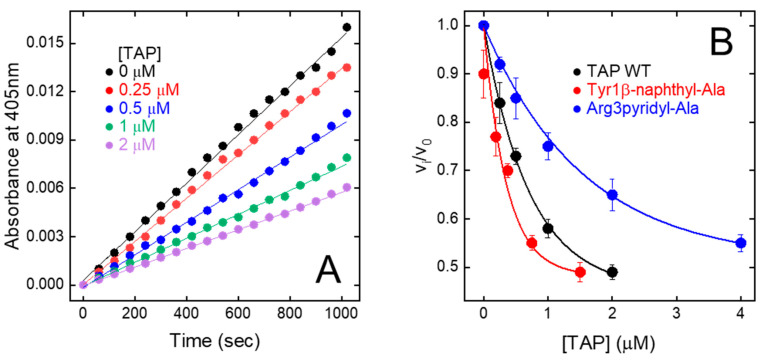
Inhibition of FXa amidolytic activity by wild-type synthetic TAP and mutated analogues. (**A**) Progress curves of pNA release by fXa. FXa solutions were pre-incubated (30 min) with increasing concentration of TAP and reaction was started by addition of chromogenic substrate RGR-pNA. Measurements were carried out at 25 °C in TBS, pH 7.4, containing 0.2 M NaCl and steady-state velocities of pNA release were determined from increase in absorbance at 405 nm. (**B**) Plot of relative velocities (v_i_/v_0_) of pNA release as function of increasing concentrations of wild-type and synthetic TAP analogues, as indicated. Notably, v_i_ and v_0_ are steady-state velocities of RGR-pNA hydrolysis in presence and absence of inhibitor, respectively. Data points are average of three independent measurements, with error bars as ±SD. As relevant examples, only fXa inhibition properties of Tyr1β-naphthyl-Ala and Arg3pyridyl-Ala analogues are reported. Data points were analysed according to the competitive tight-binding inhibition model, to yield K_I_ values reported in Table 1.

The relative initial rate of substrate hydrolysis (v_i_/v_0_) was plotted against TAP concentration (Figure 5A). The data points were analysed within the framework of the slow-tight and competitive inhibition model [32,48], allowing for the calculation of an inhibition constant (K_I_) of 200 ± 19 pM (Figure 5B), identical to that previously determined for the natural inhibitor (K_I_ = 135–580 pM) [9,49,50]. Overall, fXa inhibition data provide a clear-cut indication that stepwise SPPS is a convenient method to produce reasonably high yields of fully functional TAP.

### 3.5. Design, Synthesis, and SAR Studies of Synthetic TAP Analogues Containing Non-Coded Amino Acids

**Figure 6:**
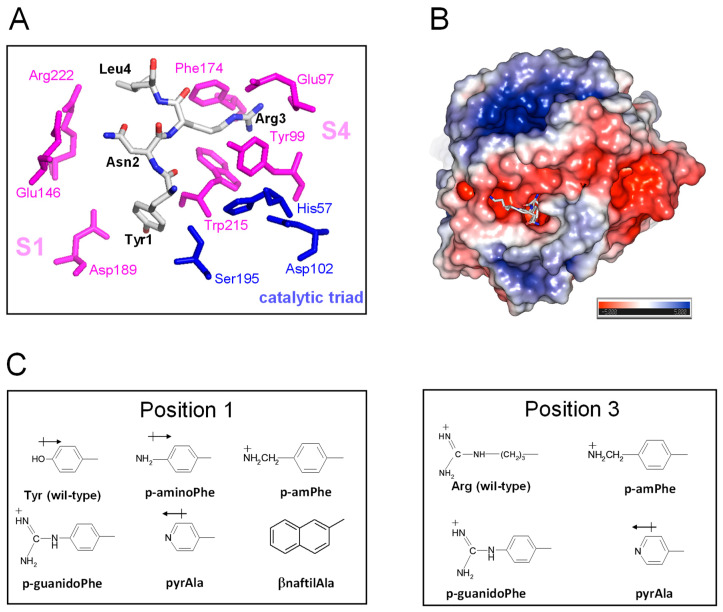
The details of the TAP-fXa interaction and the molecular structure of substituting amino acid side chains. (**A**) A close-up view of the interaction of the N-terminal amino acids 1–3 with the fXa active site. The amino acids of the TAP involved in the interaction with fXa are highlighted in stick and colour coded (carbon, grey; oxygen, red; nitrogen, blue); the catalytic amino acids of fXa are in blue, while those of the substrate specificity sites are coloured magenta. The picture was generated on the crystallographic structure of the TAP-fXa complex (1kig.pdb). (**B**) The surface electrostatic potential of fXa. The stick representation of the first three residues of TAP is also shown. The surface is coloured according to the electrostatic potential (blue, positive; red, negative) and expressed as kJ/(mol·q), as indicated. Calculations were performed using the APBS programme, run on the coordinates of the TAP-fXa complex, after removing the coordinates of TAP 4–60. Images were generated using the PyMOL ver. 1.3 Molecular Graphics System. (**C**) The structure of the substituting non-coded amino acids at position 1 and 3 of TAP.

TAP-fXa interaction. The results of kinetic [49], mutagenesis [50], and X-ray crystallographic [10] studies concurrently indicate that TAP binds to fXa in a two-step kinetic mechanism, whereby some parts of the negatively charged C-terminal α-helix (^47^Asp-Tyr^49^ and ^54^Asp-Ile^60^) interact in a slow-binding step with the positively charged “secondary” binding site on the fXa structure, comprising residues in the protease sodium binding site (Arg222′ and Lys224′) and in the autolysis loop (Arg143′, Glu146′, Lys147′, and Arg149′). This “secondary” interaction serves to induce the N-terminal residues of TAP (^1^Tyr-Arg^3^) to rearrange and to lock into the factor Xa active site that represents the “primary” binding site for TAP (Figure 6A). In particular, Tyr1 enters the primary specificity (S1) site and its -OH group is hydrogen bonded to the carboxylate of Asp189′ at the bottom of the S1 site. Arg3 penetrates into the apolar S4 site of fXa, formed by a narrow, U-shaped “cage” of aromatic amino acids (Tyr99′, Phe174′, and Trp215′). The side chain Asn2 is hydrogen bonded to the Gln192′ carboxamide group and Gly218′ carbonyl oxygen in the upper rim of the protease S1 site, and interacts intra-molecularly with Tyr49 in the C-terminal helix, likely helping to stabilise the inhibitor bioactive conformation. Interestingly, the guanidyl-group of Arg3 makes favourable orthogonal charge–quadrupole interaction with the π-electron cloud of the Trp215′ side chain and planar/stacked π-π interactions with the Tyr99′ and Phe174′ side chains [51]. The interaction of Arg3 with the fXa “aromatic cage” is further reinforced by the charge-charge interaction of Arg3 with the Glu97′ side chain, located in the upper rim of the S4 site [10] (Figure 6A).

The precise fit of TAP N-terminal amino acids into the protease recognition sites and the combination of both hydrophobic and polar (i.e., hydrogen bonding, charge-charge, charge-π, and π-π) interactions are the major determinants of the extraordinary affinity and specificity of TAP for fXa. This interaction has been optimised by evolution such that even conservative amino acid substitutions result into a dramatic reduction in the affinity of TAP for fXa [50,52]. For instance, despite the electronegative nature of the S1 site calling for a positively charged amino acid at P1, the replacement of Tyr1 with Arg leads to a 100-fold drop in affinity, whereas Tyr1Asp or Tyr1Glu mutations (both introducing a negative charge) reduce affinity only five-fold [52]. Likewise, the substitution of Asn at position 2 with a small neutral (i.e., Ala) or charged (i.e., Asp or Arg) amino acid reduces affinity by approximately 1350- to 24,050-fold. Finally, the replacement of Arg3 with Lys or Asn leads to a drop in the affinity of TAP for fXa by approximately 1150- and 42,000-fold, respectively [50]. Altogether, these observations indicate that the chemical integrity of the N-terminal tripeptide sequence in TAP is mandatorily required to support high-affinity binding with fXa.

Selection of amino acid substitutions and SAR studies on TAP 1–60. Protein engineering with non-coded amino acids allows the introduction of non-coded amino acids with “tailored” side chains into proteins and to finely “tune” the physicochemical of proteins site-specifically, thus allowing for the effective transfer of the SAR approach, typical of modern medicinal chemistry on small molecules, to the protein level.

Starting from the analysis of the crystallographic structure of the TAP-fXa complex and considering that even subtle changes in the TAP N-terminal region can dramatically reduce the inhibition of fXa (see above), here we exploited the relative facility with which the wild-type inhibitor has been synthesised in the fully bio-active form and the versatility of SPPS to conduct SAR studies aimed at identifying the properties responsible for the exceedingly high affinity of TAP for fXa and to eventually obtain even more potent TAP analogues. Hence, we produced eight analogues of TAP 1–60 in which Tyr at position 1 or Arg at position 3 were replaced with non-coded amino acids (Figure 6B). The FXa primary specificity (S1) site is electronegative (Figure 6C), as it contains the negatively charged Asp189′ that favourably couples with positive chemical entities present in both FXa substrates (e.g., RGR-pNA) and small-molecule inhibitors [10]. Following the principle of minimal structural frustration, Tyr1 was replaced with non-coded amino acids retaining the aromatic ring of Tyr and containing in the *para*-position functional groups of increasing basicity, i.e., NH_2_- (*p*-amino-Phe) < pyridyl-Ala < NH_2_-CH_2_- (*p*-aminomethyl-Phe) < NH_2_-[C=NH]-NH- (*p*-guanido-Phe). Notably, Tyr1 was also replaced by β-naphthyl-Ala to probe the effect of a larger and more hydrophobic amino acid on the interaction of TAP with the S1 site. Next, the apolar S4 binding site, representing the other key hot spot for the TAP binding on fXa, was probed by replacing the Arg side chain at position 3 of TAP with that of pyridyl-Ala, *p*-aminomethyl-Phe, or *p*-guanido-Phe (Figure 6B). Whereas all these amino acids retain the positive charge of Arg3, their side chains are more conformationally rigid than the long and flexible side chain of arginine. TAP analogues were synthesised as described above for the wild-type protein, subjected to disulphide oxidative refolding, purified, characterised for their chemical identity by HR-MS (Supplementary Table S1), and finally tested for fXa inhibitory potency. The results of enzyme inhibition assays are reported in Table 1, along with the relevant physicochemical properties of the substituting amino acid side chains.

Tyr1 → X. Due to the mesomeric effect, p-amino-Phe is not protonated (pK_A_ = 5.08) at the solution pH at which enzyme inhibition assays were performed (i.e., pH 7.4). The hexocyclic –NH_2_ group has a partial positive charge and essentially retains the hydrogen bonding capacity of the Tyr –OH group, without introducing any significant steric constrain. As a result, the corresponding TAP analogue (Tyr1*p*-amino-Phe) is only slightly (r = 0.9) less potent than wild-type TAP. The replacement of Tyr1 with pyridyl-Ala results in a two-fold reduction in the inhibitory potency of TAP. As for *p*-amino-Phe, even pyridyl-Ala is not protonated at pH 7.4, but the more electronegative nitrogen in the pyridyl ring is partially negatively charged and this could destabilise interaction with the electronegative Asp189 in the S1 site. Contrary to the results of mutagenesis studies showing that the substitution of Tyr1 with Arg leads to a 100-fold reduction in affinity, the presence of an aromatic amino acid carrying a net positive charge results in only a modest increase (i.e., *p*-aminomethyl-Phe, r = 1.1) or decrease (*p*-guanido-Phe, r = 0.54) in the affinity of TAP for fXa, suggesting that steric, electronic, and orientation effects also play a role in inhibitor binding, beyond simple electrostatic coupling. Interestingly, the replacement of Tyr1 with the bulkier and more hydrophobic β-naphthyl-Ala enhanced the inhibitory potency of TAP (r) by >2.5-fold (Table 1 and Figure 5B). Modelling studies indicate that the β-naphthyl-group is properly oriented to extensively fill the S1 cavity, making favourable van der Waals interactions with Ala190′ and Val213′ and quadrupole–quadrupole (π-π) interaction with the aromatic ring of Tyr228′ (Supplementary Figure S2).

Arg3 → X. The data in Table 1 indicate that the perturbation of position 3 with the uncharged pyridyl-Ala only slightly reduced inhibition (r = 0.6), whereas the presence of the positively charged p-aminomethyl-Phe and p-guanido-Phe resulted in a remarkable loss in the affinity of TAP for fXa by 130- and 80-fold, respectively. This loss is, however, much smaller than that measured in earlier mutagenesis studies after conservative Arg3→Lys mutation, i.e., a 1150-fold decrease [50]. Notably, *p*-aminomethyl-Phe and *p*-guanido-Phe can be considered as conformationally rigid analogues of Lys and Arg, respectively. Whereas the “freezing” of side chain rotamers could enhance affinity by reducing the entropy loss of binding, it could also orient the hexocyclic charged groups in a sub-optimal conformation (Supplementary Figure S2). Our findings once more indicate that evolution selected Arg at position 3 of TAP as the best amino acid for interacting with the stereochemically constrained S4 site of fXa. In fact, the long and flexible Arg side chain can adapt to and productively interact with the “aromatic cage” in the S4 site, exploiting different physicochemical interactions (e.g., π-π and charge-π), and concomitantly harbour the carboxylate-group of Glu97′.

## 4. Discussion

Blood coagulation is a finely orchestrated process involving a cascade of enzymatic reactions, in which haemostatic pathways are activated in response to vascular injury, and involves three distinct events, i.e., vascular constriction, platelet activation and aggregation, and final clot formation [53,54]. Importantly, the dysregulation of one or more of these processes is at the basis of the onset of thrombotic diseases which are most often characterised by the aberrant generation of thrombin and, due to their heavy social and economic burden, currently represent a global health problem. Thrombotic diseases frequently appear in clinics as the expression of coagulative complications variably occurring in different (apparently unrelated) diseases, such as type-2 diabetes, chronic kidney disease [55], inflammatory bowel disease [30], cancer [56], rheumatoid arthritis [57], autoimmune diseases [31], amyloidosis [33], and bacterial [30,58] and viral [59] infections.

Historically, the pharmaceutical repertoire for anticoagulant therapy that has been developed during the last 50 years was essentially restricted to heparin and dicoumarol derivatives [60], which act (albeit with different mechanisms) as “indirect” inhibitors of thrombin generation and function, while their use in therapy requires careful monitoring and is often associated with major side effects [60,61]. More recently, the introduction in clinical practise of small-molecule inhibitors of thrombin and fXa as Direct Oral Anticoagulants (DOAC) significantly improved the safety profile of anticoagulants, providing effective anticoagulation with reduced bleeding risk, even though frequent side effects are still a concern [12,62,63].

Both thrombin and fXa are chymotrypsin-like serine proteases playing a pivotal role in haemostasis. Thrombin is the final effector protease of the coagulation cascade, as it proteolytically converts soluble fibrinogen into insoluble fibrin aggregates and activates platelets by cleaving type-1 protease-activated receptor on the surface of platelets, which become bio-adhesive and agglutinate to form, together with fibrin, the final thrombus structure [54]. Interestingly, thrombin is generated after proteolysis of prothrombin zymogen by fXa in the prothrombinase complex, which is formed in the presence of calcium ions by fXa and fVa on the negatively charged phospholipid surface of activated platelets. The rate of prothrombin activation by fXa in the prothrombinase complex is approximately 10^6^-fold faster than that of fXa alone. Given the unique position of fX in the coagulation pathway and its critical role as the effector of thrombin generation, fXa has emerged as an attractive target for the development of new anticoagulants of both synthetic and natural origin [5].

The specific modulation of vertebrate blood clotting has been mastered by hematophagous invertebrates that have evolved highly effective mechanisms to facilitate the acquisition of a blood meal via the production of exquisitely potent and selective inhibitors of the proteases involved in the blood coagulation cascade [1,2,3,4,5]. The approval of the natural hirudin HV1 variant from the leech *Hirudo medicinalis*, along with its recombinant derivatives lepirudin (Refludan^®^) and desirudin (Revasc^®^/Iprivask^®^) and the synthetic bivalent analogue bivalirudin (Angiomax ^®^), as direct thrombin inhibitors for anticoagulant therapy newly stimulated the resurgence of pharmaceutical interest in natural products isolated from haematophagous organisms for developing new anticoagulants [5,64,65]. Among novel anticoagulants of natural origin, TAP inhibition of fXa is safer than the blockage of thrombin, as fXa is considered the “gatekeeper” of blood coagulation and its inhibition does not impair the generation of sufficient amounts of thrombin to sustain physiological haemostasis. The efficacy of TAP and its safer pharmacological profile has been proven either for the isolated inhibitor [12,13,14,15,16,17,18] and, more recently, when it is fused to single-chain antibodies to localise TAP anticoagulant activity at the thrombus level [19,20,21,22].

From this viewpoint, here we have undertaken the total chemical synthesis, disulphide oxidative renaturation, and a thorough chemical, conformational, and functional characterisation of wild-type TAP. After the peptide chain assembly was completed, crude reduced R-TAP was allowed to fold at pH 8.3 under mildly denaturing conditions (0.5 M Arg) and, counterintuitively, under reducing conditions (250 µM β-mercaptoethanol), allowing for the achievement of a renaturation yield of >85% (Figure 2C). This yield is remarkably high, especially if one considers that, for a polypeptide chain containing six Cys residues that form three S-S bonds, the statistical probability of ending up with the native disulphide topology is 1 in 15 (i.e., <7%). Noteworthy, the presence of arginine in the refolding buffer significantly improved folding yields, likely because the mild chaotropic/denaturing properties of the Arg side chain guanido group enhances the solubility of those folding intermediates that, otherwise, would have precipitated during TAP folding. Furthermore, the addition of a reducing agent, such as β-mercaptoethanol, was found to be beneficial, allowing the preferential reduction of scrambled misfolded S-S bonds that were less stable to reducing agents than native disulphides, thus giving the polypeptide chain additional chances to fold into the more stable native S-S bond topology [29]. The overall final yield of fully active (K_I_ = 186 pM) N-TAP, comprising the peptide chain assembly, disulphide renaturation, and purification (>98%), was estimated as ~20%, which is remarkably high considering (i) the length of the TAP chain (i.e., 60 amino acids), (ii) the concomitant presence of synthetically challenging amino acids along the polypeptide sequence (i.e., five Arg and two Trp residues), and (iii) the presence of six Cys residues that should form three S-S bonds with a unique native-like topology. Notably, much lower yields (0.1–4%) for the synthesis of other disulphide cross-linked peptides of similar size have been recently reported [66,67].

While the spectroscopic characterisation of TAP supports the native-like structure of the synthetic inhibitor, HDX-MS measurements reveal that, despite the similarity of the three-dimensional fold and S-S bond topology of TAP and BPTI, the TAP structure is by far more flexible than that of BPTI (Figure 4), in keeping with the 20 °C lower melting temperature of TAP compared to BPTI [27]. In the following, we discuss how the difference in protein dynamics could impact on both the folding and inhibitory mechanisms of the two proteins.

**Figure 7:**
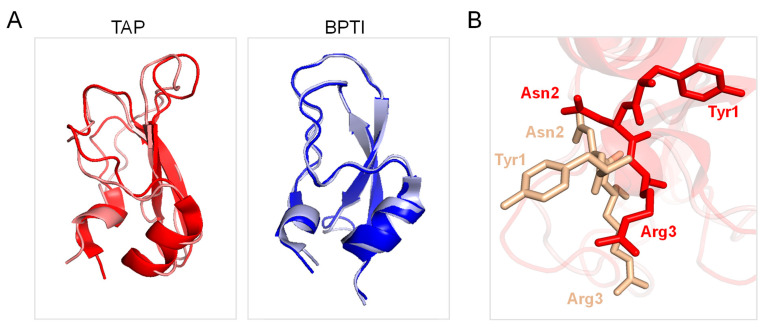
Comparison of the three-dimensional structure of TAP and BPTI in the free and proteases-bound form. (**A**) Ribbon drawing representation of the superposition of the NMR solution structure of free TAP (1tcp, red) with that of TAP in the crystallographic structure of the fXa-TAP complex (1kig, light gold). Ribbon drawing representation of the superposition of the NMR solution structure of free BPTI (1pit, blue) with that of BPTI in the crystallographic structure of the trypsin-BPTI complex (4y0y, light grey). (**B**) Close-up view of the superposition of the conformation of TAP sequence Tyr1-Asn2-Arg3- in the free (1tcp, red) and fXa-bound state (1kig, light gold).

Careful kinetic investigations indicated that disulphide oxidative renaturation of BPTI occurs through very few, stable intermediates displaying native-like S-S bond topology, whereas the same folding process for TAP involves the formation of many nearly isoenergetic and rapidly interconverting intermediates possessing non-native S-S bonds [24]. It is likely that, under the experimental conditions of the folding reaction tested in this study, the enhanced conformational flexibility of the TAP native structure would allow the easier reduction and re-shuffling of S-S bonds with a resulting higher disulphide heterogeneity in the folding intermediates, which however converge to the more stable native structure. Another key aspect that differentiates TAP from BPTI is that the latter inhibits trypsin and other serine proteases (i.e., trypsin, chymotrypsin, elastase, plasmin, and kallikrein) with a lock-and-key mechanism [27], whereas TAP specifically blocks fXa function by exploiting an induced fit (adaptive) mechanism [10]. In particular, BPTI inserts the reactive-site loop, RSL, (13Pro-Arg17) in the protease active site according to a rigid-body mechanism through which the amino acids in the RSL align in an antiparallel manner with the 214Ser-Gly216 β-strand of the protease active site via electrostatic coupling of Lys15 with Asp189′ in the enzyme primary (S1) specificity site. Notably, Cys5 of BPTI (forming an S-S bond with Cys55) helps to lock the RLS in the bioactive conformation for productive enzyme inhibition, but neither the inhibitor nor the protease changes their conformation upon macromolecular complex formation (Figure 7A, *right panel*) [68]. Conversely, TAP displays almost absolute specificity for fXa and inhibits the protease via a kinetic two-step process, whereby the negatively charged C-terminal helix first electrostatically couples with a positive patch (i.e., the secondary binding site) on the fXa structure and then orients the N-terminal TAP residues Tyr1 and Arg3 into the S1 and S4 recognition sites of the protease, respectively [10,49,50]. From the comparison of the TAP structure in the free/unligated [25,38] and fXa-bound form [10], it can be concluded that the inhibitor binds to the cognate protease with an induced-fit mechanism, as after binding to fXa, TAP undergoes a massive reorganisation in the N-terminal tetrapeptide orientation, along with changes in the Cys5-Cys59 bond conformation (Figure 7A, right panel). The specificity and inhibitory potency of TAP for fXa is therefore the result of a delicate balance between favourable interactions where the inhibitor engages with the protease active site and the energetic cost that it has to pay to overcome the energy barrier existing between the “native” conformation that TAP assumes in the free/unligated form and the “bioactive” conformation it explores in fXa-bound form. Intriguingly, the enhanced conformational flexibility TAP appears to have been “evolutionarily engineered” to lower the energetic cost of TAP adaptation to fXa active site. Conversely, the more rigid structure that BPTI assumes in solution is already well suited for trypsin binding in a lock-and-key mechanism [68]. Our interpretation is strongly supported by the solution NMR structure of the inhibitor, showing that, in contrast to what observed in the NMR structure of BPTI [69], the N-terminal tripeptide of TAP has only a few NOE distance constraints and that all the aromatic amino acids undergo rapid flipping motions about the Cβ-Cγ bonds [25], indicative of both locally and globally enhanced flexibility, respectively, of TAP compared to BPTI [70].

Hence, the TAP/BPTI pair represents a nice example of divergent evolution, whereby the two inhibitors could derive from a common Kunitz-type ancestor protein module into which nature has evolved different properties (e.g., amino acids types and main-chain dynamics) for modulating protease targeting and inhibitor potency. A closely resembling trend has been observed with ubiquitin [71] and the Thi-S protein [72]. These two proteins display only 14% sequence identity and totally different functions. Ubiquitin is involved in the targeted degradation of proteins in eukaryotes, whereas Thi-S is a sulphur carrier protein that plays a central role in thiamin biosynthesis in prokaryots. Nevertheless, they share an identical structural fold [72]. The stereochemical degeneracy, observed for the TAP/BPTI pair, makes TAP an attractive system for studying fundamental processes in proteins (e.g., sequence-folding and sequence-function relationships) to possibly insert novel functionalities in the molecular scaffold of TAP that could mimic more complex systems (e.g., metal-binding sites, enzyme catalytic sites, chemotactic sequences, and protease inhibitory epitopes).

Finally, the results herein reported emphasise once more the importance of solid-phase synthesis as a convenient method to produce even longer polypeptides in high yields and highlight the possibility of exploring structure–activity-relationships (SAR) in small proteins of therapeutic interest by introducing non-coded amino acid residues with tailored side chains. The results of SAR studies indicate that there is little (if any) room for improving fXa inhibition by TAP and that only the replacement of Tyr1 with β-naphthyl-Ala moderately enhanced affinity by 2.5-fold, whereas all other substitutions left the affinity of TAP for fXa essentially unchanged or even caused a strong reduction in the inhibitory potency (Table 1). For comparison, in the hirudin–thrombin system, the substitution of a Tyr residue at position 3 of hirudin with β-naphthyl-Ala or biphenyl-Ala resulted in a 40- and 200-fold increase in the affinity for thrombin [28]. Our results are in keeping with the dramatic loss of affinity earlier observed after site-directed mutagenesis of the TAP N-terminal region with standard amino acids [50,52] and suggest that the TAP structure has been finely implemented by nature for fXa inhibition such that even subtle chemical modification can dramatically reduce affinity for the target protease. It is likely that this is the result of host–pathogen co-evolution, whereby TAP and fXa exploited both hydrophobic and polar interactions to achieve inhibition potency and selectivity.

Overall, our work highlights the sheer plasticity of the Kunitz fold, possibly allowing the insertion of novel functionalities (e.g., metal-binding sites, enzyme catalytic sites, chemotactic sequences, protease inhibitory peptidyl epitopes) in the molecular scaffold of TAP, specifically in the inter-cysteine ‘‘loops’’, via the relatively facile synthetic strategy herein reported. Even though TAP has not yet been approved for clinical use, our data could represent a good starting point to design novel synthetic analogues with improved profiles.

## Figures and Tables

**Table 1:** Inhibition of FXa amidolytic activity by the synthetic wild type and Tyr1 → X and Arg3 → X analogues of TAP ^a^.

TAP Analogues	K_I_ (pM) ^b^	r ^c^	vdW Volume (Å^3^) ^d^	Log P ^e^	μ (Deybe) ^f^	pKa ^g^
**Tyr1 (wild-type)**	**186 ± 5**	**1.00**	**138**	**1.97**	**−1.57**	**10.5**
Tyr1*p*-amino-Phe	210 ± 7	0.89	109	1.39	−1.84	4.63
Tyr1*p*-aminomethyl-Phe	165 ± 4	1.13	165	−0.80	−0.39	9.34
Tyr1*p*-guanido-Phe	343 ± 8	0.54	180	0.17	-	10.88
Tyr1pyridyl-Ala	352 ± 12	0.53	122	1.74	−2.26	5.25
Tyr1β-naphthyl-Ala	71 ± 3	2.62	180	4.00	0.30	-
**Arg3 (wild-type)**	**186 ± 5**	**1.00**	**148**	**−0.06**	**-**	**12.0**
Arg3*p*-aminomethyl-Phe	2.4·10^4^ ± 5·10^2^	0.008	165	−0.80	11.11	9.34
Arg3*p*-guanido-Phe	1.5·10^4^ ± 3·10^2^	0.012	180	0.17	-	10.88
Arg Arg3pyridyl-Ala	290 ± 7	0.64	122	1.74	−2.26	5.25

^a^ FXa inhibition assays were conducted as detailed in the Methods. ^b^ The values of the equilibrium inhibition constants, K_I_, were obtained as described in the Methods. ^c^ r is the ratio between the Ki value of the wild-type TAP and that of the synthetic analogue; ^d,e^ van der Waals (vdW) volume and LogP values of the organic molecules corresponding to the amino acid side chains, where P is the octanol/water distribution constant. ^f^ μ is the electric dipole moment of the amino acid side chains. ^g^ pKa values of the Tyr side chain and of the acid conjugated form of basic amino acids at position 1 and 3.

## Data Availability

All other data that support the findings of this study are available from the corresponding author upon reasonable request.

## Supplementary Materials

The following supporting information can be downloaded at https://www.mdpi.com/article/10.3390/biomimetics9080485/s1, Figure S1: Determination of S-S bond topology of N-TAP by peptide mass fingerprint analysis; Figure S2: Molecular modelling of TAP analogues interaction with FXa active site; Table S1: Mass spectrometry data and molar absorptivity values of TAP analogues produced in this study; Table S2: Mass spectrometry data of the peptides obtained by mass fingerprint analysis.
