# Supplementary material for: Chemical Synthesis and Structure–Activity Relationship Studies of the Coagulation Factor Xa Inhibitor Tick Anticoagulant Peptide from the Hematophagous Parasite Ornithodoros moubata
Source: Biomimetics (Basel). 2024 Aug 12;9(8):485. doi: 10.3390/biomimetics9080485 (PMC11351495; doi:10.3390/biomimetics9080485)
Supplement: Supplementary file 1 [file biomimetics-09-00485-s001.zip › biomimetics-3124002-supplementary.pdf]

SUPPLEMENTARY MATERIALS

Chemical Synthesis and Structure-Activity Relationship Studies of the Coagulation Factor Xa Inhibitor Tick Anticoagulant Peptide from the Hematophagous Parasite *Ornithodoros moubata*

Vincenzo De Filippis [1,] *, Laura Acquasaliente [1], Andrea Pierangelini [1], Oriano Marin [2]

[1] Laboratory of Protein Chemistry & Molecular Haematology, Department of Pharmaceutical and Pharmacological Sciences, School of Medicine, University of Padova. [2] Department of Biomedical Sciences, School of Medicine, University of Padova.

*** To whom correspondence should be addressed**: Prof. Vincenzo De Filippis, Department of Pharmaceutical and Pharmacological Sciences, School of Medicine, University of Padua, via Marzolo 5, Padua, 35131 Italy. Phone: (+39) 0498275698, e-mail: vincenzo.defilippis@unipd.it

Table S1. Mass spectrometry data and molar absorptivity values of the synthetic TAP analogues produced in this study.

TAP	MW (experimental) [a] a.m.u	MW (theoretical) a.m.u.	Molar absorptivity [b] (ε) at 280nm $M^{-1} \cdot cm^{-1}$
WT	6978.3 ± 0.2	6978.6	18,140
Tyr1*p*-aminomethyl-Phe	6979.3 ± 0.5	6979.0	16,860
Tyr1*p*-amino-Phe	6991.4 ± 0.3	6991.6	16,860
Tyr1*p*-guanido-Phe	7020.6 ± 0.6	7019.7	16,860
Tyr1pyridyl-Ala	6963.8 ± 0.2	6963.6	16,860
Tyr1β-nafthyl-Ala	7012.9 ± 0.3	7012.6	22,240
Arg3*p*-aminomethyl-Phe	6999.2 ± 0.1	6999.0	18,140
Arg3*p*-guanido-Phe	7026.8 ± 0.4	7026.6	18,140
Arg3pyridyl-Ala	6970.9 ± 0.7	6970.4	18,140

[a] The values reported refer to the average masses; [b] the values were calculated utilizing molar absorption coefficients of 1280 M^{-1} $cm^{-1}$for Tyr, 5690 M^{-1} $cm^{-1}$for Trp, 120 M^{-1} cm^{-1} for Cys and 5380 M^{-1} cm^{-1} for βnaftilAla

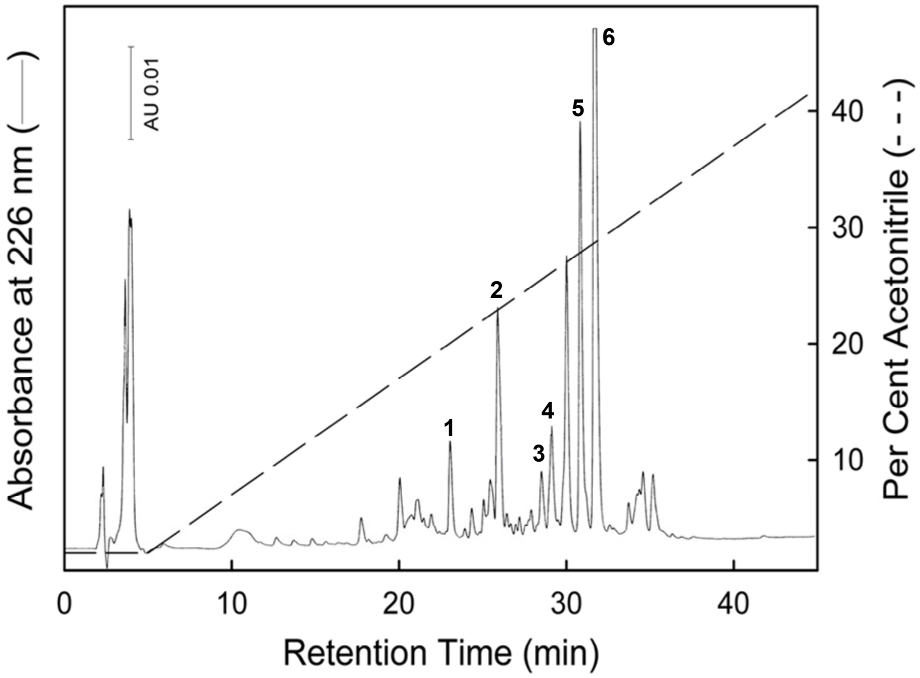

Supplementary Figure S1. Determination of S-S bond topology of N-TAP by peptide mass fingerprint analysis. Purified N-TAP was treated (4 h at 40°C) with trypsin and chymotrypsin, immobilised on Sepharose-4B gel. After filtration, the proteolysis reaction was analysed by LC-MS/MS, and the chemical identity of the material eluted in correspondence of the numbered peaks is reported in the **Supplementary Table S2**.

Table S2. Mass spectrometry data of the peptides obtained by mass fingerprint analysis.

Peak	Sequence	MW (experimental) [a] a.m.u	MW (theoretical) a.m.u.
1	^{57}NACI^{60}<br>^{5}CIKP^{8}	1032.7 ± 0.3	1032.0
2	^{24}AYF^{26}	400.2 ± 0.1	400.2
3	^{31}GGCDSF^{36}<br>^{53}RDCF^{56}	1120.3 ± 0.5	1121.6
4	^{31}GGCDSF^{36}<br>^{54}DCF^{56}	965.5 ± 0.2	964.4
5	^{9}RDWTDECDSNEGGER^{23}<br>^{37}WICPEDHTGADY^{48}	3027.9 ± 0.6	3027.2
6	^{9}RDWTDECDSNEGGER^{23}<br>^{37}WICPEDHTGADYY^{49}	3189.6 ± 0.3	3189.6

[a] The values reported refer to the average masses.

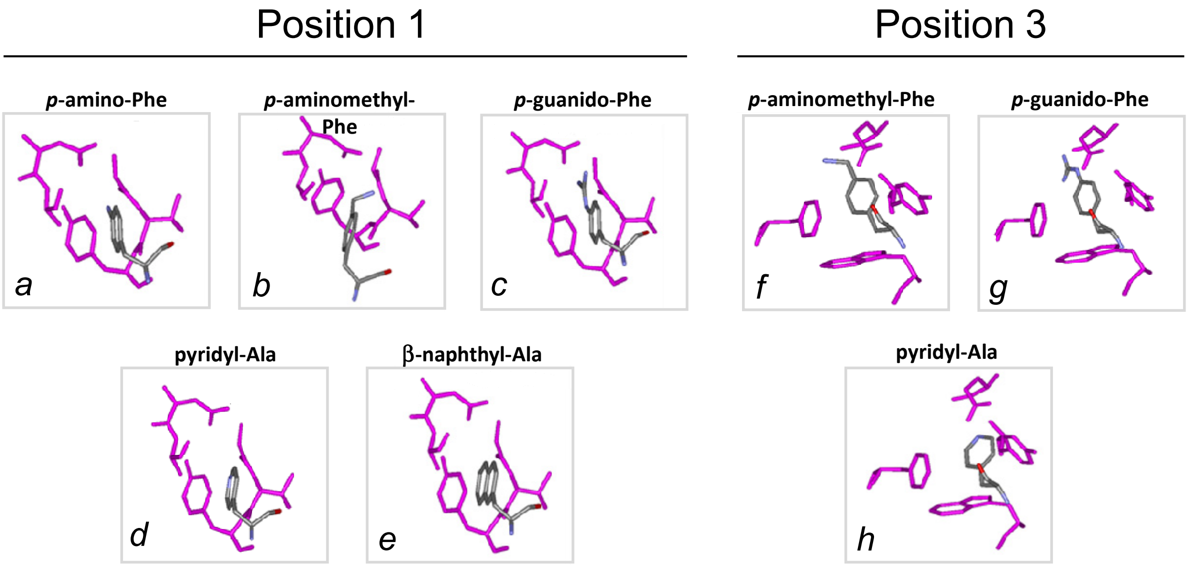

Supplementary Figure S2. Molecular modelling of TAP analogues interaction with FXa active site. The structure of the synthetic TAP analogues (color coded) in the fXa-bound (magenta) state was modelled using the MOE ver. 15 software, run on the crystallographic structure of TAP-fXa complex (lkig.pdb).
